# Double Trouble: Association of Malignant Melanoma with Sporadic and Genetic Forms of Parkinson’s Disease and Asymptomatic Carriers of Related Genes: A Brief Report

**DOI:** 10.3390/medicina59081360

**Published:** 2023-07-25

**Authors:** Christos Koros, Athina-Maria Simitsi, Anastasia Bougea, Nikolaos Papagiannakis, Roubina Antonelou, Ioanna Pachi, Efthalia Angelopoulou, Andreas Prentakis, Athena Zachou, Chrysa Chrysovitsanou, Ion Beratis, Stella Fragkiadaki, Dionysia Kontaxopoulou, Efthymia Eftymiopoulou, Evangelia Stanitsa, Constantin Potagas, Sokratis G. Papageorgiou, Efstratios Karavasilis, Georgios Velonakis, Vasilios Prassopoulos, Xenia Geronicola-Trapali, Leonidas Stefanis

**Affiliations:** 1 1st Department of Neurology, Eginition Hospital, National and Kapodistrian University of Athens, 11528 Athens, Greece; simitsh@yahoo.gr (A.-M.S.); annita139@yahoo.gr (A.B.); nickpap88@gmail.com (N.P.); rantonelou@gmail.com (R.A.); pachiioanna@gmail.com (I.P.); angelthal@med.uoa.gr (E.A.); a.zachou@hotmail.com (A.Z.); chrysachrysovitsan@gmail.com (C.C.); ionas96@hotmail.com (I.B.); st.fragkiadaki@gmail.com (S.F.); d.kontaxopoulou@hotmail.com (D.K.); faih.efthymiopoulou@gmail.com (E.E.); eva.st.92@gmail.com (E.S.); cpotagas@otenet.gr (C.P.); sokpapa@med.uoa.gr (S.G.P.); lstefanis@bioacademy.gr (L.S.); 2 Nuclear Medicine Unit, Attikon Hospital, 12462 Athens, Greece; a_prentakis@yahoo.gr (A.P.); xeniatr@yahoo.gr (X.G.-T.); 3 Research Unit of Radiology, 2nd Department of Radiology, Medical School, National and Kapodistrian University of Athens, “Attikon” University General Hospital, 11528 Athens, Greece; stratoskaravasilis@yahoo.gr (E.K.); giorvelonakis@gmail.com (G.V.); 4 Nuclear Medicine Unit, IASO Hospital, 15123 Athens, Greece; vprasopoulos@gmail.com

**Keywords:** Parkinson’s disease, malignant melanoma, Leucine Rich Repeat Kinase 2, Glucocerebrosidase, genetic

## Abstract

*Introduction:* Previous epidemiological evidence has established the co-occurrence of malignant melanoma (MM) and Parkinson’s disease (PD). Shared molecular mechanisms have been proposed to be implicated in this relationship. The aim of the present study was to assess the prevalence of MM in patients with sporadic and genetic types of PD, as well as in asymptomatic carriers of PD-related genes. *Methods:* Data regarding past medical history and concomitant disease of 1416 patients with PD (including 20 participants with prodromal disease who phenoconverted to PD), 275 healthy controls (HCs) and 670 asymptomatic carriers of PD-related genes were obtained from the database of the Parkinson’s Progression Markers Initiative (PPMI). Focus was placed on information about a medical record of MM. We also retrieved data regarding the genetic status of selected PPMI participants with a positive MM history. *Results:* In total, 46 patients with PD reported a positive MM history. Concerning the genetic forms of PD, nine of these PD patients (2.47%) carried a Leucine Rich Repeat Kinase 2 (LRRK2) gene mutation (mainly the G2019S), while eight (4.49%) harbored a Glucocerebrosidase (GBA) gene mutation (mainly the N370S). No alpha-synuclein (SNCA) gene mutation was identified in patients with an MM history. The remaining 29 PD patients (3.5%) were genetically undetermined. In total, 18 asymptomatic carriers of PD-related genes had a positive medical history for MM: among them, 10 carried an LRRK2 gene mutation (2.69%) and 10 a GBA gene mutation (3.51%) (2 were dual carriers). MM history was identified for seven HCs (2.5%). *Conclusions:* We replicated the previously reported association between genetically undetermined PD (GU-PD) and MM. A correlation of LRRK2 mutations with the development of MM could not be verified in either symptomatic PD patients or asymptomatic carriers, implicating distinct pathogenetic mechanisms as compared to GU-PD. Importantly, despite the limited literature evidence on Gaucher disease, this study highlights for the first time the relatively high prevalence of MM among asymptomatic and symptomatic PD GBA mutation carriers, with potential clinical implications.

## 1. Introduction

Previous epidemiological evidence indicates a relationship between Parkinson’s disease (PD) and cancer. In general, PD patients appear to be relatively protected against some types of cancer and it has been suggested that PD is rarer in cancer patients (inverse comorbidity). However, despite the fact that PD patients exhibit a relatively decreased risk of the development of most types of cancers, this is not the case for some specific malignancies. PD has been linked to increased risks of breast cancer, brain cancer, malignant melanoma (MM) and non-melanocytic skin cancer, but decreased bladder, prostate and colon cancer risks. Genetic and environmental factors like smoking are crucially implicated in this interaction between these two distinct disorders [1,2].

Literature data from epidemiological studies support an association of PD with MM. Using Rochester Epidemiology Project records, PD patients have been reported to present a 3.8-fold higher risk of pre-existing melanoma compared to healthy controls (HCs) and melanoma patients have been reported to present a 4.2-fold higher probability of PD [3]. In another report in Israel, melanoma incidence in PD patients was 4.4 higher than in the general population [4]. A large nationwide study was conducted in the Korean population to evaluate the risk of developing skin cancer in PD patients. According to this study, PD patients displayed a higher risk of skin cancer. In particular, older male PD patients (>65 years), had a greater risk of melanoma than HCs [5]. In the National Institutes of Health (NIH) Exploratory Trials in PD (NET-PD) Long-term Study 1 (LS-1) cohort, the risk for MM was increased compared to that reported in the general population, and it was similar to the risk mentioned in previous clinical trial cohorts [6]. In the DATATOP clinical trial, the standardized event ratio was 3.3, indicating that MM incidence was higher than expected [7].

A recent meta-analysis assessing the link between PD and polymorphisms related to cancers, using cross-phenotypes polygenic risk score analytical methods, also confirmed the already reported genetic correlation between PD and MM [8]. Nevertheless, no large studies have been conducted in genetic PD, and this is important, because it may shed light on the association between the two disorders.

**Figure 1:**
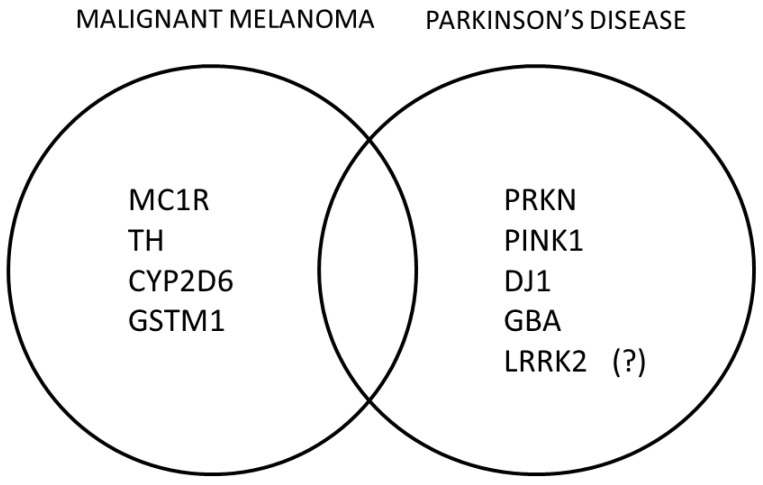
MM- or PD-related genes reported to play a role in the crosstalk between these distinct disorders. (?) The role of LRRK2 in the development of MM is uncertain.

Melanoma represents the most aggressive form of skin cancer; it originates from melanocytes, which produce melanin, the pigment that colors hair, skin and the iris of the eye. Melanoma is associated with red hair and fair skin, which is the phenotype of the (melanocortin 1 receptor) MC1R gene polymorphisms [9]. Dopaminergic neuron and melanocytic cells have a shared origin in the neural crest (neuroectodermal cells) and melanogenesis capability [10]. Common biochemical pathways or shared risk factors might account for this connection. Immune dysregulation has been proposed to be highly involved in the neurodegenerative process of dopaminergic neurons and the development of tumors by melanocytes. L-dopa represents a substrate for the process of synthesis of both melanin in melanocytes and dopamine in dopaminergic neurons. Alterations in melanin or dopamine synthesis enzymes, including tyrosinase (TYR) and tyrosine hydroxylase (TH), may also contribute to increased vulnerability to both melanoma and PD. Interestingly, alterations in some low-penetrance genes like vitamin D receptor possibly elevate the risk for both melanoma and PD [11] (Figure 1).

It is notable that a number of PD-related genes may also predispose individuals to melanoma (Figure 1). Such genes are either mutated in melanoma cells, or they encode proteins implicated in the development of MM [10]. A-synuclein (α-syn), the main protein associated with PD, has been suggested to be involved in some types of neoplasms, thus suggesting a potential molecular link between melanoma and PD [12]. α-Syn is also able to interact with both TH and TYR [13]. Interestingly, 129Ser-phosphorylated α- is abundantly found in melanoma cells, but not in normal skin [10].

The Parkinson’s Progression Markers Initiative (PPMI) is an observational, prospective natural history study funded by the Michael J. Fox Foundation and other partners. More than 50 sites in different countries are participating. PPMI assesses the progression of clinical (motor and non-motor) deficits, with a particular focus on cognitive impairment. Moreover, additional biomarkers studied include imaging, biologic fluid and genetic markers, as well as digital data of PD. The main target of PPMI is to identify markers of disease progression in order to facilitate therapeutic PD trials. PPMI clinical enrollment includes prodromal, recently diagnosed PD participants, genetic forms of PD and healthy controls.

The aim of the present study was to assess the prevalence of MM in patients with sporadic and genetic types of PD, as well as asymptomatic carriers of PD-related genes in the PPMI study. The merits of using PPMI data include the international nature of the study, the long-term follow up and the prerequisite of positive DATSCAN that ensures accurate PD diagnosis. Additional advantages of PPMI are the systematic homogeneous collection of data, the existence of carefully selected controls and the fact that, at least for non-genetic PD, the patients, when recruited, are at an early unmedicated PD stage. Notably, regarding our current assessment of melanoma in genetic PD patients, the in-depth genetic analysis taking place in PPMI enables to evaluate separately genetically undetermined PD (GU-PD) from specific genetic forms.

## 2. Methods

The protocol of the PPMI study is adequately described on the study website (www.ppmi-info.org, (accessed on 31 December 2022)). PD participants eligible for enrolling in PPMI comprise de novo patients with less than 2 years of disease duration who have not received dopaminergic treatment before their participation in the study. Moreover, PD patients carrying a pathogenic mutation are also eligible regardless of treatment status. For carriers of LRRK2 and GBA pathogenic variants, the disease duration before enrollment should be less than 2 years. Previous dopaminergic therapy is allowed for this cohort. In contrast, PD carriers of mutations in rare genes like SNCA, PRKN and PINK1 are able to participate in PPMI regardless of disease duration. All patients should have basal ganglia dopaminergic denervation exemplified by an abnormal DATSCAN SPECT imaging in order to be able to enroll in PPMI.

Healthy controls (HCs) participating in PPMI should have no current significant neurological disorder, no first-degree relative with PD and normal dopamine transporter DATSCAN SPECT imaging.

The Prodromal cohort includes individuals at risk for PD. Participants are selected based on risk factors or clinical features related to PD (REM-Sleep Behavior Disorder RBD, Hyposmia as measured by the UPSIT test and known genetic risk variants). There is an age limit of 60 years, with the exception of carriers of rare genetic variants. No clinical diagnosis of parkinsonism or dementia is permitted.

Patients are followed longitudinally with a full-length clinical visit taking place annually and a shorter site visit and/or telephone visit every 6 months. Epidemiological data are collected during the initial visits. Participants undergo a complete physical and neurological examination during each follow-up visit. Past medical history and information on concomitant diseases and medication are collected as well. A number of clinical assessments, including motor evaluation with the Movement Disorder Society–sponsored Unified Parkinson’s Disease Rating Scale (MDS-UPDRS) II, III and IV, also take place. Non-motor symptoms are examined by means of MDS-UPDRS I and I-Patient Questionnaire, olfaction using the University of Pennsylvania Smell Identification Test (UPSIT), daytime sleepiness using the Epworth Sleepiness Scale and REM sleep behavior disorder (RBD) using the RBD questionnaire. Autonomic dysfunction is evaluated using the Scales for Outcomes in Parkinson’s Disease–Autonomic questionnaire (SCOPA-AUT) scale, depression by means of the Geriatric Depression Scale (GDS), stress using the State-Trait Anxiety Inventory (STAI) and compulsive behaviors using the Questionnaire for Impulsive-Compulsive Disorders in Parkinson’s Disease (QUIP). Moreover, a detailed neuropsychological assessment is performed by experienced neuropsychologists (The Montreal Cognitive Assessment (MoCA), the Hopkins Verbal Learning Test (HVLT), the Benton Judgement of Line Orientation test, the Letter Number Sequencing Test (LNST), the Symbol Digit Modalities Test (SDMT), semantic and phonemic verbal fluency, the Boston Naming Test and the Trail Making Test). Further, questionnaires on motor or cognitive competence are also completed by participants. Laboratory assessments also take place, including the collection of biological fluids (blood, urine, cerebrospinal fluid) as well as brain imaging (Magnetic Resonance Imaging (MRI) and DAT SPECT). Analysis of the aforementioned specimens and notably genetic testing for pathogenic PD-related variants is a cornerstone of the PPMI study.

Data were obtained on 31 December 2022 from the database of the PPMI (www.ppmi-info.org/access-data-specimens/download-data), RRID:SCR_006431. Up-to-date information on PPMI is available at www.ppmi-info.org. Data regarding past medical history and concomitant disease of 1416 PD patients (including 20 participants with prodromal disease who phenoconverted to PD), 275 healthy controls (HCs) and 670 asymptomatic carriers of PD-related genes were downloaded on 31 December 2022. Available data from PD or HC patients who were screened and/or enrolled in the PPMI study were assessed (including PD patients who had only undergone a screening visit, as long as past medical history, concomitant disorders and genetic data were available). The present study was conducted in agreement with the principles of the Declaration of Helsinki. Signed informed consent was obtained from all participants recruited. The study was approved by the Scientific Board of all PPMI sites involved (including the Scientific Board of Eginition hospital).

For the purpose of this study, focus was placed on information regarding a medical history of MM. Additional epidemiological data including race, sex and age at PD or MM diagnosis were also collected. We also retrieved data on the genetic status of selected PPMI participants with a report of MM history (carriers of the Leucine Rich Repeat Kinase 2 (*LRRK2*) gene mutations, mainly the G2019S, Glucocerebrosidase (*GBA*) gene mutations, mainly the N370S, and alpha-synuclein (*SNCA*) gene mutations, mainly the p.A53T).

## 3. Results

In total, 46 PD patients had a positive history for MM out of 1416 PD patients screened and/or enrolled in PPMI (including 20 prodromal participants who phenoconverted to PD). The majority were men (72%) with a mean age at MM diagnosis of 59.6 ± 10.8 years and a mean age at PD diagnosis of 67.5 ± 7 years. In most cases MM was antecedent to PD diagnosis (78%). All participants in the study with a positive MM history were of white race, and 12.5% were of Askenazi Jewish ancestry (Table 1). Smoking habits were known for only 9 (out of 46) PD patients with a positive MM history (4 smokers/5 non-smokers).

We focused on PD subgroups according to their genetic background and a positive history of MM. Overall, 830 PD patients were genetically undetermined (GU-PD) and 29 patients in this group had a history of MM (3.5% of the GU-PD cohort). Concerning the genetic PD forms, 365 PD patients carried a pathogenic *LRRK2* mutation (mainly the G2019S) and 9 of these participants had a positive history of MM (2.47% of LRRK2 PD). Interestingly, 178 PD patients harbored a *GBA* mutation (mainly the N370S) and 8 of them reported MM in their medical records (4.49% of GBA PD). No dual LRRK2 and GBA symptomatic PD carriers with a history of MM could be identified. Furthermore, despite the fact that 31 PPMI PD patients carried pathogenic *SNCA* mutations (mainly the p.A53T), none had a history of MM. Notably, no mutation carrier of other rare recessive PD-related genes *(PRKN, PINK1)* had a history of MM. We should also point out that, among 275 HC participants in the study, a positive MM history was identified for 7 HCs (2.5%) (Table 2).

We also assessed asymptomatic carriers of PD-related genes (684 participants). In total, 18 asymptomatic carriers of PD-related genes reported a medical history for MM. Specifically, 372 PPMI participants were asymptomatic carriers of a pathogenic *LRRK2* mutation and a positive MM history was reported for 10 of them (2.69% of the asymptomatic LRRK2 cohort). Additionally, 285 participants were asymptomatic carriers of a pathogenic *GBA* mutation and 10 had a positive MM history (3.51% of the asymptomatic GBA cohort). We should highlight the fact that two of the asymptomatic participants with a positive MM record were dual carriers of LRRK2 and GBA mutations. In contrast, none of the 14 asymptomatic SNCA mutation carriers had a positive MM history (Table 2).

## 4. Discussion

We were able to replicate the previously reported prevalence of MM in PD patients. Many different cohorts of GU-PD, as well as both symptomatic and asymptomatic carriers of PD-related genes, have been studied. Our results regarding a relatively increased percentage of GU-PD with a positive MM history confirms the association, although the difference with the controls is not particularly marked, as reported in other epidemiological studies. This observation indicates that GU-PD cases (not linked to specific genetic defects) are in fact associated with MM. We should highlight an important limitation related to the relatively small number of PD patients in the PPMI subgroups as compared to previous large epidemiological studies of MM occurrence assessment in sporadic PD. Due to this fact, no meaningful statistical comparisons could be performed between the various PD subgroups and controls. Moreover, the GU-PD cohort in PPMI included de novo PD patients with a disease history of less than two years upon enrollment. This has its advantages, as it excludes the possibility that the process of PD clinical manifestations, including the administration of rasagiline or dopaminergic drugs such as L-Dopa, could be driving this association. Notably, data in our study are derived from the PPMI database and are based on past medical history as provided by patients and caregivers. As a result, some information may be missing as compared to data derived from a national health registry entered by healthcare professionals during regular clinical assessment. However, the PPMI study is rich in genetic PD forms, while large epidemiological studies lack this information and mainly focus on idiopathic PD patients irrespectively of their genetic status. A final limitation was that we were unable to investigate the possible effects of environmental factors, such as sun exposure or smoking, which are known risk factors for MM and PD. Data such as sun exposure have not been collected in the PPMI study, while smoking habits were recorded only remotely through the FOUND sub-study (by means of a phone call or e-mail) and this information is missing in many PPMI participants. We were able to access data regarding smoking habits for only 9 (out of 46) PD patients with a positive MM history (4 smokers/5 non-smokers).

The association between PD and MM may result from multiple interactions between genetic and environmental factors. The recessive PD genes involved in mitochondrial function and the regulation of oxidative stress play a role in both disorders (Figure 1). Impaired expression of the Parkin gene (*PRKN*) that is implicated in tumor suppression may enhance the growth of certain tumors. A previous study showed an important role for *PRKN* as a tumor suppression factor both in melanoma risk and progression [14]. *PINK1* can have an oncogenic or a tumor suppression function and *DJ-1* overexpression has been linked to some neoplasms like melanoma, breast and lung cancers [15].

The main focus of our study was the impact of the genetic background (mostly involving relatively frequent autosomal dominant PD genes like *LRRK2* and *GBA*) on the possibility of developing MM. *LRRK2* gene mutations are among the most frequent genetic causes of familial PD, but also cause some sporadic PD cases. LRRK2 promotes aggregation of α-synuclein into Lewy bodies and tau tangles and is also implicated in mitochondrial function, oxidative stress and neuroinflammation. In addition to brain tissue, *LRRK2* is also expressed in peripheral tissues including lung, gut, blood cells and breast. This observation may explain the increased colon, breast, brain and hematological malignancies in *LRRK2* PD, further highlighting the connection between *LRRK2* mutations and cancer [10,16]. Our results assessing PPMI data indicate that, in *LRRK2* carriers, symptomatic or asymptomatic, the prevalence of MM is no different from that in the controls. A plausible explanation would be that the epidemiological–biological factors connecting GU-PD to MM do not apply to *LRRK2* carriers. Literature data regarding MM in LRRK2 PD are rather contradictory. A meta-analysis by Lee and co-authors (2022) showed that PD patients carrying *LRRK2* gene mutations displayed a decreased risk of lung and colorectal cancer cancers and a higher risk of brain cancer and melanoma [1]. Literature data indicate that carriers of the G2019S mutation in *LRRK2* have an increased cancer risk (including blood, brain and breast cancer). Melanoma incidence in PD patients carrying *LRRK2* gene mutations who had a heterozygous p.Gly2019Ser pathogenic variant was similar or even higher than the risk of the development of melanoma among patients with idiopathic PD [16,17]. On the other hand, according to a previous epidemiological study, PD patients with the *LRRK2* G2019S mutation displayed a greater global cancer risk [10,16]. MM was not among the cancer types that have been firmly linked to *LRRK2* mutations like breast cancer in women [16], colon and skin malignancies or leukemia [17]. In the study of Ruiz-Martinez and co-authors, there was no specific relationship between the R1441G or G2019S LRRK2 gene mutations and any form of malignancy [18]. Finally, a study in an Ashkenazi Jewish patient cohort with PD assessed the association of a single missense mutation (G2019S) in the *LRRK2* gene with MM. Only 1.2% of the MM patients carried the mutation, rendering the link of *LRRK2* PD to MM uncertain [19].

An important finding of our study was the association of MM with *GBA* mutations. GBA-PD patients seem to have higher rates of MM, and this possibly also applies to the asymptomatic state (the difference from the controls is not particularly marked in this cohort). It is highly probable that the same factors that connect GU-PD to MM may apply to GBA-PD, a fact that is aligned with the low penetrance of GBA mutations. Regarding asymptomatic carriers, this is indeed an association that may be of special interest, if replicated in other studies, as it is known that few of these will develop PD, and very few (if any, based on the study of Simuni and co-authors using PPMI data (2020), with only 3% showing DATSCAN deficit) are in a state of prodromal PD [20]. The above might indicate a PD-independent but GBA-related connection. Literature evidence on MM in *GBA* carriers is scarce, with the exception of limited reports on Gaucher disease (GD) patients carrying homozygous or compound heterozygous *GBA* mutations. It has been shown that deficits in *GBA* enzyme activity, as in Gaucher disease, are related to a higher melanoma risk. The occurrence of other neoplasms including skin (basal cell carcinoma, squamous cell carcinoma), multiple myeloma and other hematological malignancies, breast, pancreatic carcinoma, prostate cancer, thyroid cancer, glioblastoma, colon, lung, renal cell and sarcoma was also increased in GD patients [21]. In the study by Taddei et al. in an Ashkenazi Jewish population, including 367 GD patients—54% of whom were homozygous for the N370S GBA mutation—the risk of developing cancer and hematologic malignancies during a long-term follow-up was demonstrated to be increased. In particular, there was a higher risk of MM development (OR 2.26) [22]. Similarly, in 1525 male adult US veterans with GD, the risk of MM was also increased (OR 3.07) [23]. Except for previous data on GD, our study is the first to underline the relatively high prevalence of MM in PD patients harboring heterozygous *GBA* mutations (without systemic GD involvement) and notably in asymptomatic *GBA* mutation carriers.

A number of shared pathways have been suggested in order to explain the association of MM in carriers of pathogenic *GBA* mutations. The role of undegraded substrates like β-glucosylceramide (GlcCer) and β-glucosylsphingosine (GlcSph), and the subsequent alterations in the immune and inflammatory responses, appear to be the most relevant hypotheses regarding the molecular pathway linking PD patients carrying GBA mutations to MM. GlcCer- and GlcSph-derived sphingolipids may be responsible for the increased risk of developing hematological disorders and cancers like MM that are associated with GD. It has been shown that sphingosine 1-phosphate has oncogenic properties, such as immune cell trafficking, inflammation and tumor progression [24]. Glucocerebrosidase deficits have also been linked to impaired autophagy, which is a regulator of cell survival. Deficits in autophagy result in increased tumor susceptibility by means of mitochondrial dysfunction and the production of reactive oxygen species [25]. Finally, protein misfolding and the impairment of a-synuclein degradation, which has been shown to be a hallmark in *GBA* mutations related to PD, can also trigger a pathway leading to cancer development, including malignant melanoma [26].

Although there is previously published work regarding idiopathic PD and MM, the novelty of our present report is the assessment of genetic PD forms (*LRRK2, GBA* and *SNCA* mutation carriers). Such literature data are very scarce for *LRRK2*, while the only existing literature on the association between *GBA* and melanoma was focused on Gaucher disease patients. We used data from the PPMI database with a considerable number of participants and a uniform means of data collection. Additionally, the current report is the first to assess asymptomatic carriers of PD-related genes (*LRRK2* and *GBA*), who have not been evaluated previously.

It appears that, apart from the already established co-occurrence of GU-PD and MM, patients harboring PD-related mutations in genes like *GBA* (but not *LRRK2*), as well as asymptomatic *GBA* mutation carriers, are at risk of developing MM, with possible clinical implications in therapeutics and monitoring (including the need for frequent skin examination by an expert dermatologist). Furthermore, the elucidation of shared molecular pathways leading to the development of PD or MM might pave the way for novel preventive or disease-modifying treatments.

## 5. Conclusions

In the present study, we replicated the previously reported association between GU-PD and MM. Regarding genetic PD forms, a correlation of LRRK2 mutations with the development of MM could not be verified in either symptomatic PD patients or asymptomatic carriers, implicating distinct pathogenetic mechanisms as compared to GU-PD. Notably, despite the limited literature evidence on Gaucher disease, this report highlights for the first time the relatively high prevalence of MM among asymptomatic and symptomatic PD GBA mutation carriers, with potential clinical implications. 

## Figures and Tables

**Table 1:** Demographic and clinical data of PD patients with a positive MM history.

Feature	
Race	100% White (of which 12.5% Askenazi Jewish ancestry)
Sex	72% Male
MM antecedent to PD	78% of cases
	Mean ± SD
Age at MM diagnosis	59.6 ± 10.8 yo
Age at PD diagnosis	67.5 ± 7 yo

**Table 2:** Absolute number and % percentage of a positive MM history in PD patients (idiopathic or genetic forms) and asymptomatic carriers of PD-related genes.

N of PPMI Participants with A Positive History of MM	Genetically Undetermined GU-PD	Genetic Background LRRK2+	Genetic Background GBA+	Dual Carriers LRRK2+/GBA+
N of PD PATIENTS (46/1416)	29/830 (3.5%)	9/365 (2.47%)	8/178 (4.49%)	0
N of Asymptomatic Carriers of PD-RELATED Mutations (18/670)	-	8 + 2/372 (2.69%)	8 + 2/285 (3.51%)	2 (included in LRRK2+ and GBA+)
N of Healthy Controls (7/275)	7/275 (2.5%)	0	0	0

## Data Availability

Data used in this study have been downloaded from the PPMI database and are available following reasonable request and a sign-up process.
